# Measures of Sleep-Related Fears in Children: A Systematic Review of Psychometric Properties Using COSMIN

**DOI:** 10.1007/s10567-025-00526-6

**Published:** 2025-05-21

**Authors:** Melissa Aji, Xiaomin Xu, Emma A. McDermott, Madeline Metz, Annabel Songco, Maddison O’Gradey-Lee, Chloe Y. S. Lim, Gemma Sicouri, Laura Parrish, Jennifer L. Hudson

**Affiliations:** 1 Black Dog Institute, University of New South Wales, Hospital Rd, Randwick NSW 2031, Sydney, NSW Australia; 2 Faculty of Medicine and Health, University of New South Wales, Sydney, NSW Australia; 3 University of North Carolina at Chapel Hill, Chapel Hill, USA; 4 School of Psychology, University of New South Wales, Sydney, NSW Australia

**Keywords:** COSMIN, Children, Measures, Sleep, Anxiety, Review

## Abstract

Sleep-related fears (i.e. fears related to sleep, nighttime and/or occurring before sleep) are linked to the development of anxiety and sleep problems in children. Parent- and child-report measurement tools are key to increasing understanding and facilitating better identification for treatment to prevent disorder development. We conducted a systematic review with the aim of identifying the breadth of parent- and child-report measures that include an assessment of sleep-related fears in children from 7 to 12 years old (stage 1) and reviewing the psychometric properties of measures that more comprehensively assess sleep-related fears (i.e. 3 or more items) using the COSMIN checklist (stage 2) to provide recommendations for measures (stage 3). A systematic search was performed using MEDLINE, EMBASE, ERIC and PsycINFO in accordance with PRISMA guidelines. At stage 1, we retrieved 14,495 records. Of these, 66 papers met eligibility and included 43 distinct measures. At stage 2, 11 out of the 43 measures met criteria for more comprehensive measurement of sleep-related fears. Findings demonstrated mixed and incomplete data and very little high-quality evidence. No measures met criteria for recommendation and the majority of measures require further validation studies.

**Supplementary Information:**

The online version contains supplementary material available at 10.1007/s10567-025-00526-6.

## Introduction

Insomnia and related sleep disturbances are common in children, with prevalence rates of up to 40% (Combs et al., 2016; Fricke-Oerkermann et al., 2007; Lunsford-Avery et al., 2021; Owens et al., 2000a, 2000b). School-aged children (aged 7–12 years) with sleep problems can face many deleterious outcomes, including emotional and behavioural difficulties (Gregory & O’Connor, 2002), reduced quality of life (Combs et al., 2016; Magee et al., 2017), poor academic performance (de Zambotti et al., 2018), increased risk-taking behaviours (de Zambotti et al., 2018) and negatively impacted social and family functioning (Meltzer & Mindell, 2007). Chronicity of sleep disturbance in children ranges from 15 to 60% (Fricke-Oerkermann et al., 2007; Zhang et al., 2011). When left untreated, sleep problems are more likely to persist and tend not to resolve with age, particularly amongst children with elevated anxiety (Gregory et al., 2005; Simola et al., 2014).

Anxiety and sleep difficulties are highly comorbid. Up to 90% of children with an anxiety disorder experience at least one sleep-related problem, with as many as 82% reporting multiple sleep-related problems (Alfano et al., 2007; Chase & Pincus, 2011; Storch et al., 2008). Moreover, the majority of anxious children (76–85%) experience clinically significant sleep disturbance (Alfano et al., 2010; Weiner et al., 2015). Sleep disturbances are associated with the development of later internalising difficulties and higher levels of anxiety severity (Alfano et al., 2007; Quach et al., 2018).

A common manifestation in children that intersects anxiety and sleep problems is nighttime fears. These fears are heterogeneous and can include bedtime fears, separation fears, darkness, personal security and imagination-based fears at night (Gordon et al., 2007; King et al., 1997; Mooney, Graziano & Katz, 1985). With respect to related externalising behaviours, children may exhibit bedtime resistance, reassurance seeking, co-sleeping and effortful attempts at sleep (Cortesi et al., 2008; Muris et al., 2001a, 2001b, 2001c). Although co-sleeping can be related to parental preferences and cultural traditions (Owens, 2004), co-sleeping in the context of children with sleep-related problems tends to emerge from anxiety and sleep disturbances (Cortesi et al., 2008; Palmer et al., 2018).

Nighttime fears impact up to 80–85% of children aged 7–12 years (Muris et al., 2001a, 2001b, 2001c) and can serve as early predictors of sleep disturbances and anxiety disorders (El Rafihi-Ferreira et al., 2019; Kushnir & Sadeh, 2011). Given the temporal nature of nighttime fears, children with these fears are particularly vulnerable to developing sleep problems. The pre-sleep period is frequently linked to heightened anxiety and higher levels of cognitive activity and worry in adults with anxiety (Bélanger et al., 2005). Pre-sleep arousal is a significant component of both cognitive and neurocognitive models of persistent sleep disturbance and insomnia (Akerstedt et al., 2007; Espie, 2002; Harvey,  2002; Perlis et al., 1997; Spielman et al., 1987). Moreover, studies on adults have demonstrated a link between pre-sleep arousal and sleep problems (Espie, 2002; Wicklow & Espie, 2000). There is emerging evidence to suggest this can be generalised to youth (Alfano et al., 2009; Gregory & Eley, 2005; Gregory et al., 2010). In children, pre-sleep cognition has been shown to be associated with decreased total sleep duration and overall increased sleep disturbance (Alfano et al., 2009, 2010).

In this review, ‘sleep-related fears’ refers to any fears or worries associated with sleep, nighttime and/or the pre-sleep period. This may include fears related to content, such as the dark, separation at night, imaginary creatures or burglars (i.e. nighttime fears). It may include worries that occur before bedtime, including generalised worry before bed. It can also include both cognitions and behavioural manifestations, such as avoidance and safety behaviours (e.g. bedtime resistance or reassurance seeking at night). This term was selected for its broad scope in encompassing both nighttime fears and pre-sleep arousal.

There is a need to increase our understanding of sleep-related fears in children to enable better and more accurate measurement and identification of specific targets for intervention and prevent disorder development. Consistency in the use of high-quality measures can enhance the accuracy of treatment targets for sleep, anxiety and nighttime fears in children (Aslund et al., 2018; Galgut et al., 2024; Lewis et al., 2021; Wang et al., 2017). Questionnaires are the most commonly used measure for sleep-related fears and provide a practical and feasible method of assessment. Yet there is a lack of knowledge and inconsistency in the use of measurement tools, compromising research conclusions and advancement in the field (Lewis et al., 2021). To fully capture the multifaceted nature of sleep-related fears, questionnaires should assess the content, severity, frequency and behavioural manifestations of these fears.

This review focuses on children aged 7–12 years, as this age group experiences the highest frequency and severity of nighttime fears, compared to other age groups (Gordon et al, 2007; Muris et al., 2001a, 2001b, 2001c). Additionally, these children are in a vulnerable period where the occurrence of sleep problems is linked with increased mental health problems in early adolescence (Cooper et al., 2023), reiterating the importance of assessment and treatment of sleep-related problems in this age group. The current review also focuses on measures that have been validated in English.

The aim of this systematic review is to identify the breadth of parent- and child-report measures that include an assessment of sleep-related fears in children from 7 to 12 years old. The second aim is to review the psychometric properties of measures that more comprehensively measure sleep-related fears to ultimately recommend a small battery of robust tools.

## Methods

This review was prepared and conducted in accordance with the Preferred Reporting Items for Systematic Reviews and Meta-Analyses (PRISMA) and the COnsensus-based Standards for the selection of health Measurement INstruments (COSMIN) (Page et al., 2021; Prinsen et al. 2018). The protocol was registered with PROSPERO (registry number CRD42024567073). This review was conducted through the following three sequential stages:

- Stage 1: Systematic literature search of anxiety and sleep measures which include item(s) that assess sleep-related fears in children from 7 to 12 years old.
- Stage 2: Evaluation of the methodological quality for articles and quality of measurement properties in a subset of measures that assess sleep-related fears using the COSMIN Risk of Bias Checklist.
- Stage 3: Recommendations for a battery of measures for sleep-related fears in children.

## Stage 1: Systematic Literature Search

### Search Strategy

A systematic search of MEDLINE, EMBASE, ERIC and PsycINFO was undertaken from inception to March 2024. The search strategy was developed with a university librarian and piloted through multiple preliminary searches. Four search categories were developed: (1) constructs pertaining to sleep-related fears (e.g. anxiety, fear or sleep), (2) measurement (e.g. questionnaire, scale), (3) psychometrics (e.g. validity, reliability) and (4) children (e.g. paediatric, youth). See Appendix 1 for an example of the search strategy. Reference lists of included papers and prior review articles were hand-searched to identify additional records.

### Eligibility Criteria and Study Selection

Studies were included if (1) the mean age of the sample of children fell between 7.00 and 12.00 years inclusive; (2) the measure had the main aim of assessing mental health broadly or measuring constructs related to sleep-related fears (i.e. anxiety, fear, worry, or sleep); (3) the measure had at least one item related to sleep-related fears; (4) the main purpose of the paper was to describe the development of or examine psychometric properties of the measure; (5) responses were reported by parent or child and (6) the study was published in a peer-reviewed journal.

Studies were excluded if (1) the tool had the main purpose of measuring unrelated anxieties and phobias (e.g. social anxiety, panic disorder, obsessive–compulsive disorder), as they are unlikely to thoroughly explore fears related to sleep or sleep conditions other than sleep disturbances or insomnia; (2) the study population of interest was children with neurodevelopmental or medical comorbidities, e.g. autism, attention-deficit/hyperactivity disorder and epilepsy; (3) the measure was used in a medical or hospital setting; (4) the measure was in a language other than English; (5) they were not original research, i.e. reviews, books, meta-analyses, expert opinions, conference abstracts, case studies and dissertations and (6) the paper was in a language other than English. Measures in a language other than English were excluded due to the author’s proficiency being limited to English, as this review required examining item content in detail.

Titles and abstracts were double-screened independently by 10 reviewers (MA, EM, JH, AS, MO, CL, GS, XX, LP, MM) using Covidence (2024). Any disagreements were resolved through discussion and consensus. All full-text papers were screened by the lead author (MA). Forty-three per cent of full-text papers were double-screened independently by 2 other reviewers (XX, MM). Percentage and kappa agreement at full text were 97% and 0.85, respectively, indicating almost perfect agreement. Disagreements were resolved through joint discussion.

### Data Extraction

Data extraction was completed on all included articles by lead author (MA). Descriptive characteristics extracted included citation, year published, name of measure, informant (parent or child), total number of items, subscales, items relating to sleep-related fears and response options.

## Stage 2: Evaluation of Measurement Properties and Methodological Quality

### Eligibility Criteria and Procedure

A subset of measures was evaluated for measurement properties and methodological quality. Studies were included in this evaluation if the measure had 3 or more items that assessed sleep-related fears. For measures that consist of multiple subscales, only subscale(s) containing these relevant items were included in the analysis. As per the COSMIN checklist guidelines, each subscale was considered to be a unique measure and therefore, measurement property ratings may be different for each subscale. Hereafter, ‘measure’ will denote the subscale of interest for this paper.

This evaluation consisted of 4 steps using the COSMIN checklist: (1) assessment of the methodological quality of studies using the COSMIN Risk of Bias checklist, (2) rating studies for measurement properties using the quality criteria, (3) summarising the results of all studies for each measure, and (4) grading the quality of evidence using the GRADE (Grading of Recommendations, Assessment, Development and Evaluations) approach.

The lead author (MA) independently reviewed and rated information according to the COSMIN checklist. Before the full-scale evaluation, the COSMIN checklist was piloted on a subset of 10 studies to ensure familiarity and consistentcy in application. Any uncertainties during this process were resolved in consultation with senior author (JH).

## Stage 2: COSMIN-Guided Evaluation

### Assessment of the Methodological Quality of Studies

The COSMIN Risk of Bias Checklist was used to evaluate the methodological quality of the included studies. This was rated using a series of items including design requirements and preferred statistical methods and were rated on a 4-point scale consisting of “very good”, “adequate”, “doubtful” and “inadequate”. An overall methodological quality score was obtained using the ‘worst score counts’ method, where the lowest rating across the items for a given attribute is used.

For measurement development studies, the total quality score comprised (1) general design requirements, (2) concept elicitation and (3) pilot testing/interview study.

### Rating the Measurement Properties of Studies

The results of each study are rated against criteria for good measurement properties as sufficient ( +), insufficient (−) or indeterminate (?). These properties include content validity, structural validity, internal consistency, cross-cultural validity/measurement invariance, reliability, measurement error, hypothesis testing for construct validity and responsiveness.

Content validity studies assess the relevance, comprehensiveness and comprehensibility of a measure. Criterion validity was not assessed in this review because there is no gold standard parent- and child-report comparison for measures of anxiety or sleep. Hypotheses for construct validity (convergent and known groups) were developed by lead author (MA) and reviewed in consultation with senior author (JH).

### Summarising Results of All Studies for Each Measure

Results were summarised across multiple studies on a measurement property for each measure. Results were categorised as sufficient ( +), insufficient (−), inconsistent ( ±) or indeterminate (?). In comparison to the previous step which focused on single studies, the current step focuses on the measure, which may compromise aggregating the results of multiple studies.

### Grading the Quality of Evidence

In this final step, the evidence was graded using Grading of Recommendations Assessment, Development and Evaluation (GRADE) to determine the overall quality of the studies for each measure. Ratings were “high”, “moderate”, “low” or “very low”. The GRADE approach starts with the assumption that the evidence is of high quality. The quality of evidence is downgraded by one or two levels if there is risk of bias, inconsistency, indirectness or imprecision.

### Recommendations for a Battery of Measures for Sleep-Related Fears in Children

The recommendations for measures were based on combining overall results for each psychometric property and evidence quality. The recommendations were classified into three categories by the first author (MA): (A) most suitable (i.e. measures with high-quality evidence for sufficient content validity (any level) and at least low-quality evidence for sufficient internal consistency); (B) measures that have potential but require further validation studies (i.e. measures categorised not in (A) or (C)) and (C) measures that should not be recommended (i.e. measures with high-quality evidence for an insufficient psychometric property) (Mokkink et al., 2018).

## Results

### Stage 1: Systematic Literature Search

**Fig. 1:**
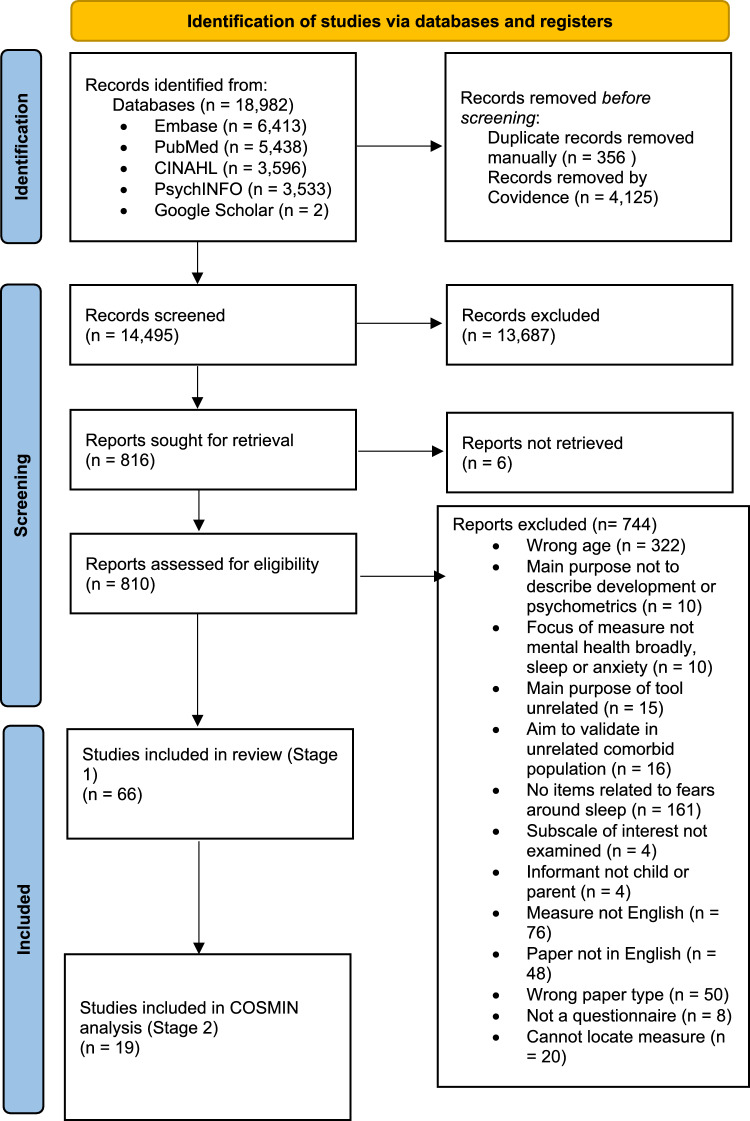
PRISMA Flow Diagram

The results of the search strategy are presented in Fig. 1, in accordance with the PRISMA guidelines (Page et al., 2021). We initially retrieved 14,495 records. Following duplicate removal and abstract and title screening, 816 studies were assessed for full-text review, which resulted in the inclusion of 66 studies.

## Stage 2a: Overview of Measures

Of the 66 papers, 43 distinct parent- and child-report measures that include any assessment of sleep-related fears were identified (Tables 1 and 2). At this stage, measures were considered distinct if they pertained to different informants (i.e. child and parent versions) and varied in length. Of these 43 measures, the majority had the main purpose of assessing anxiety or fear (*n* = 28), broader mental health (*n* = 8) and sleep (*n* = 7). Measures were informed by child report (*n* = 24) and parent report (*n* = 19). Ten measures included both parent- and child-report versions.

**Table 1:** Overview of included measures – Sleep (Stage 1)

Measure	Informant	Total items	No. of sleep-related fear items	Relevant items	Response options
Behavioural Evaluation of Disorders of Sleep Scale (BEDS) (Schreck et al., 2003)	Parent	28	3	Is afraid of noises at night; is afraid to fall asleep; needs a night light to fall asleep	5-point scale: 0 = never; 1 = almost never; 2 = sometimes; 3 = almost always; 4 = always
Children’s Report of Sleep Patterns—Child (CRSP-C) (Meltzer et al., 2013; Popper Cordts, 2016)	Child	60	4	*When you are trying to fall asleep at night:* Are you thinking about that day or the next day which makes it hard to fall asleep? Are you scared? Are you upset or worried? Is there a light on in your room (other than a nightlight)?	5-point scale: never (never happens); not very often (less than once a week); sometimes (once or twice a week); usually (3–5 times a week); always (every day)
Children’s Report of Sleep Patterns—Parent (CRSP-P) (Meltzer et al., 2013)	Parent	60	3	*When your child is trying to fall asleep at night:* Are they thinking about that day or the next day which makes it hard to fall asleep? Are they upset or worried? Are they scared?	5-point scale:(never (never happens); not very often (less than once a week); sometimes (once or twice a week); usually (3–5 times a week); always (every day)
Children’s Sleep Behaviour Scale (CSBS) (Fisher & McGuire, 1990; Fisher et al., 1989)	Parent	18	1	Has he/she expressed a fear of sleeping in the dark?	5-point scale: never (never in the past six months); rarely = (once in the past six months); occasionally (two or three times in the past six months); quite often (four or five times in the past six months); often (six times or more in the past six months)
Children’s Sleep Habits Questionnaire (CSHQ) (Owens, 2000a)	Parent	33	7	Falls asleep in own bed; falls asleep in other’s bed; afraid of sleeping alone; Struggles at bedtime; needs parent in room to sleep; afraid of sleeping in the dark; trouble sleeping away	3-point scale: usually (if the sleep behaviour occurred five to seven times/week); sometimes (for two to four times/week); rarely (for zero to one time/week)
Omnibus Sleep Problems Questionnaire (Biggs, 2012)	Parent	23	5	Fell asleep in own bed; fell asleep in others bed; needed parent in room to fall asleep; afraid of sleeping alone; moved to someone else’s bed	4-point scale: never; rarely (once per week); sometimes (2–4 times per week); usually (5–7 times per week)
Sleep Disturbance Scale for Children (SDSC) (Bruni, 1996)	Parent	26	2	The child feels anxious or afraid when falling asleep. The child goes to bed reluctantly	5-point scale: always, often, sometimes, occasionally, never

**Table 2:** Overview of Included Measures – Anxiety and Fear (Stage 1)

Measure	Construct	Informant	Total items	No. of sleep-related fear items	Relevant items	Response options
Child Anxiety Impact Scale—Child (CAIS-C) (Evans, 2017)	Anxiety	Child	27	1	*How much of your anxiety symptoms have caused problems for you in the following areas:* Sleeping at night	4-point scale 0 = not at all; 1 = just a little; 2 = pretty much; 3 = very much
Child Anxiety Impact Scale—Parent (CAIS-P) (Evans, 2017; Langley, 2004; Langley, 2014)	Anxiety	Parent	27	1	*How much of your child’s anxiety symptoms have caused problems for him or her in the following areas:* Sleeping at night	4-point scale 0 = not at all; 1 = just a little; 2 = pretty much; 3 = very much
Children’s Fear Survey Schedule (CFSS) (Ryall, 1979)	Fear	Child	48	2	The dark; bad dreams	3-point scale 0 = not (afraid) (scared) (nervous); 1 = a little (afraid) (scared) (nervous); 2 = very (afraid) (scared) (nervous)
Emotional Behaviour Scale (EBS) (Clarbour 2004)	Broad Mental Health	Child	65	1	I can’t sleep at night because I’m always thinking about my problems	2-point scale: “more like me”; “less like me”
Fear Survey Schedule for Children (FSS-FC) (Scherer, 1968)	Fear	Child	80	5	*Asked to rate the degree of fear:* Going to bed in the dark; dark places; I’m afraid of the dark; dark rooms and closets; nightmares	5-point scale:(1 = none; 2 = a little; 3 = some; 4 = much; 5 = very much
Fear Survey Schedule for Children-II (FSSC-II) (Gullone, 2000)	Fear	Child	78	1	Fear of having bad dreams	3-point scale: 1 = not scared; 2 = scared; 3 = very scared
Fear Survey Schedule for Children-Revised (FSSC-R) (Ollendick, 1983)	Fear	Child	80	4	*Asked to rate the degree of fear:* Going to bed in the dark; dark rooms or closets; dark places; nightmares	3-point scale: 1 = none; 2 = some; 3 = a lot
Fear Survey Schedule for Children-Revised Short Form (FSSC-R-SF) (Muris, 2014)	Fear	Child	25	2	*Asked to rate the degree of fear:* Going to bed in the dark; dark rooms or closets	3-point scale: 1 = none; 2 = some; 3 = a lot
Intolerance of Uncertainty Scale—Child (IUS-C) (Comer, 2009; Osmanağaoglu, 2021; Read, 2013)	Anxiety	Child	27	1	Not knowing what can happen keeps me from sleeping well	5-point scale: 1 = not at all; 3 = somewhat; 5 = very much
Intolerance of Uncertainty Scale—Parent (IUS-P) (Comer, 2009; Osmanağaoglu, 2021; Read, 2013)	Anxiety	Parent	27	1	Uncertainty keeps my child from sleeping soundly	5-point scale: 1 = not at all; 3 = somewhat; 5 = very much
Koala Fear Questionnaire (KFQ) (Muris, 2003)	Fear	Child	31	1	The dark	3-point scale: 1 = no fear; 2 = some fear; 4 = a lot of fear
MacArthur Health and Behaviour Questionnaire (HBQ) (Lemery-Chalfant, 2007)	Broad Mental Health	Parent	84	1	Scared to go to sleep without parents being near	3-point scale 1 = rarely applies; 1 = applies somewhat; 2 = certainly applies
MASC Multidimensional Anxiety Scale for Children—Child (MASC-C) (Baldwin, 2007; Grills-Taquechel, 2008; Langer, 2010; Palitz, 2018; Villabo, 2012; Wei, 2014)	Anxiety	Child	39	2	I keep the light on at night; I sleep next to someone from my family	4-point scale: 0 = never true about me; 1 = rarely true about me; 2 = sometimes true about
MASC Multidimensional Anxiety Scale for Children—Parent (MASC-P) (Langer, 2010)	Anxiety	Parent	39	2	My child keeps the light on at night; My child sleeps next to someone from my family	4-point scale 0 = never true about me; 1 = rarely true about me; 2 = sometimes true about me; 3 = often true about me
Ontario Child Health Study Emotional Behavioural Scales (OCHS-EBS) (Duncan, 2019)	Broad Mental Health	Parent	52	1	I am scared to go to sleep without my parents being near	3-point scale 0 = never or not true; 1 = sometimes or somewhat true; 2 = often or very true
Patient-Reported Outcomes Measurement Information System Paediatric (PROMIS) (Irwin, 2010)	Broad Mental Health	Child	16	1	I worried when I went to bed at night	5-point scale 0 = never; 1 = almost never; 2 = sometimes; 3 = often; 4 = almost always
Revised Child Anxiety and Depression Scale—Child (RCADS-C) (Kosters, 2015)	Broad Mental Health	Child	47	3	I feel scared if I have to sleep on my own; I worry when I go to bed at night; I would feel scared if I had to stay away from home overnight	4-point scale: 0 = never; 1 = sometimes; 2 = often; 3 = always
Revised Child Anxiety and Depression Scale—Parent (RCADS-P) (Ebesutani, 2009; Ebesutani, 2015)	Broad Mental Health	Parent	47	3	My child feels scared to sleep on his/her own. My child worries when in bed at night. My child would feel scared if he/she had to stay away from home overnight	4-point scale: 0 = never; 1 = sometimes; 2 = often; 3 = always
Revised Child Anxiety and Depression Scale (RCADS-25) (Muris, 2002a)	Broad Mental Health	Child	25	2	I feel scared if I have to sleep on my own; I worry when I go to bed at night	4-point scale: 0 = never; 1 = sometimes; 2 = often; 3 = always
Revised Child Anxiety and Depression Scale-Short Version (RCADS) (Ebesutani, 2012)	Broad Mental Health	Child	25	1	I feel scared if I have to sleep on my own	4-point scale: 0 = never; 1 = sometimes; 2 = often; 3 = always
Revised Children’s Manifest Anxiety Scale (RCMAS) (Cole, 1998; Hodges, 1990; Mattison, 1988; Pina, 2009; Reynolds, 1982; Ryngala, 2005; Scholwinski, 1985; Stavrakaki, 1991 Varela, 2006; White, 2001)	Anxiety	Child	37	1	I worry when I go to bed at night	Dichotomised: Yes/No
Revised Conners’ Parent Rating Scale—Parent (CPRS-R-P) (Conners, 1998)	Broad Mental Health	Parent	57	1	Afraid of the dark	4-point scale: 0 = not at all true; 3 = very much true
Scary Scale (Thurillet, 2022)	Fear	Child	10	1	The dark	Visual: 6 faces
Screen for Child Anxiety and Related Disorders (SCARED) – Child (Gonzalez, 2012; Hyland, 2022; Muris, 2002b; Olino, 2018; Wren, 2007)	Anxiety	Child	41	2	My child worries about sleeping alone. My child gets scared if he/she sleeps away from home	3-point scale: 0 = not true or hardly ever true; 1 = somewhat true or sometimes true; 2 = very true or often true
Screen for Child Anxiety and Related Disorders (SCARED) – Parent (Gonzalez, 2012; Hyland, 2022; Olino, 2018; Sequeira, 2020; Wren, 2007)	Anxiety	Parent	41	2	My child worries about sleeping alone. My child gets scared if he/she sleeps away from home	3-point scale: 0 = not true or hardly ever true; 1 = somewhat true or sometimes true; 2 = very true or often true
Screen for Child Anxiety Related Emotional Disorders (SCARED-66) (Muris, 1998a; Muris, 1998b; Muris, 1999; Muris, 2001a; Muris, 2001b; Muris, 2004)	Anxiety	Child	66	3	I’m afraid of the dark; I get scared when I sleep away from home; I worry about sleeping alone	3-point scale: almost never; sometimes; often
Screen for Child Anxiety Related Emotional Disorders—Revised (SCARED-R) (Muris, 2004)	Anxiety	Parent	66	3	I’m afraid of the dark; I get scared when I sleep away from home; I worry about sleeping alone	3-point scale: almost never; sometimes; often
Screen for Child Anxiety Related Emotional Disorders-Revised Parent (SCARED-RP) (Jansen, 2017)	Anxiety	Parent	69	3	I’m afraid of the dark; I get scared when I sleep away from home; I worry about sleeping alone	3-point scale: hardly ever, sometimes, or often
Separation Anxiety Avoidance Inventory (SAAI-C) (In-Albon, 2013)	Anxiety	Child	12	3	Going to sleep alone; going to sleep in my own bed; sleeping in the dark	4-point scale: 0 = never; 4 = always
Separation Anxiety Avoidance Inventory (SAAI-P) (In-Albon, 2013)	Anxiety	Parent	12	3	Going to sleep alone; going to sleep in own bed; sleeping in the dark	4-point scale: 0 = never; 4 = always
Spence Children’s Anxiety Scale—Child (SCAS-C) (Ahlen 2018; Evans, 2017; Reardon 2019; Spence, 1998)	Anxiety	Child	38	2	I feel scared if I have to sleep on my own; I am scared of the dark	3-point scale: 0 = never; 1 = sometimes; 2 = often; 3 = always (Ahlen, 2018; Reardon, 2019; Spence, 1998) 4-point scale: never; sometimes; often; always (Evans 2017)
Spence Children’s Anxiety Scale—Parent (SCAS-P) (Ahlen 2018; Evans, 2017; Reardon, 2019)	Anxiety	Parent	38		I feel scared if I have to sleep on my own; I am scared of the dark	3-point scale: 0 = never; 1 = sometimes; 2 = often; 3 = always (Ahlen 2018; Reardon, 2019) 4-point scale: never; sometimes; often; always (Evans, 2017)
Spence Children’s Anxiety Scale—Short Version (SCAS-S-C) – Child (Ahlen, 2018)	Anxiety	Child	19	1	Fear of darkness	3-point scale: 0 = never; 1 = sometimes; 2 = often; 3 = always
Spence Children’s Anxiety Scale—Short Version (SCAS-S-P) – Parent (Ahlen, 2018)	Anxiety	Parent	19	1	Fear of darkness	3-point scale: 0 = never; 1 = sometimes; 2 = often; 3 = always
The Subtle Avoidance Measure for Youth (SAMY) (Chapman, 2022)	Anxiety	Child	37	2	Sleep with family member	5-point scale: never; always
Youth Anxiety Measure (YAM-5) (Simon, 2017)	Anxiety	Child	50	1	I am afraid of the dark	4-point scale: 0 = never; 3 = always

Same author and year are differentiated by adding letters (e.g. 1998a, 1998b) after the year

There were on average two items per measure assessing sleep-related fears (range: 1–7). The item content reflected the following areas: fear of sleeping alone (32%), the dark (27%), problematic sleep behaviours (e.g. keeping light on when sleeping, requiring co-sleeping; 17%), worrying in bed (15%), fear of nightmares (5%), bedtime resistance (2%) and fear of sounds at night (1%). The majority of measures (*n* = 39) used a Likert scale ranging from 3 to 5 points. Of these, the Likert-scale response options assessed the frequency of occurrence of fears (*n* = 25), severity of fears (*n* = 11) or both (*n* = 3; e.g. ‘sometimes’ or ‘somewhat true’). Two measures used a dichotomous response scale. Two measures used a visual analogue scale measuring severity of fears, using a 3- or 6-point scale. The most commonly reported measures were the Revised Child Anxiety and Depression Scale (RCADS) Child (*n* = 10 studies) and the Multidimensional Anxiety Scale for Children—Child (MASC) (*n* = 6 studies). See Tables 1 and 2 for an overview of the measures and their features.

## Stage 2: Evaluation of Evidence and Ratings of Measurement Properties

### Summary

Of the 43 parent- and child-report measures identified in the first stage, 11 distinct measures met criteria for inclusion in stage 2, i.e. measures that more comprehensively assessed sleep-related fears (*n* = 19 papers). Table 3 provides a list of measures and studies included in stage 2. For stage 2, in accordance with COSMIN guidelines, these measures were classified as 15 distinct measures, considering each subscale separately.

**Table 3:** Included measures and studies in COSMIN Evaluation (Stage 2)

Measure	Papers
CSHQ—SA	Owens, 2000a
CSHQ- BR	
Omnibus—BA	Biggs, 2012
CRSP– C- BF	Meltzer, 2013
CRSP-C- I	
FSSC—FU	Ollendick, 1983 Muris, 2014 Scherer, 1968
BEDS	Schreck, 2003
RCADS-C-SA	Kosters, 2015
RCADS-P-SA	Ebesutani, 2009 Ebesutani, 2015
SCARED-C—SA	Muris, 1998a Muris, 1998b Muris, 1999 Muris, 2001a Muris, 2001b Muris, 2004
SCARED-C-P	
SCARED- P-SA	Jansen, 2017 Muris, 2004
SCARED-P-P	
SAAI-C	In-Albon, 2013
SAAI—P	

Same author and year are differentiated by adding letters (e.g. 1998a, 1998b) after the year

*CSHQ* children’s sleep habits questionnaire: *SA* separation anxiety, *BR* bedtime resistance, Omnibus: *BA* bedtime anxiety, *CRSP-C* children’s report of sleep patterns- child report: *BF* bedtime fears, *I* insomnia scale, *FSSC* fear survey schedule for children: *FU* fear of the unknown, *BEDS* behavioural evaluation of disorders of sleep, *RCADS* revised child anxiety and depression scale: *C-SA* child version – separation anxiety, *P-SA* parent version – separation anxiety, *SCARED* screen for child anxiety related emotional disorders, *C-SA* child version – separation anxiety, *P-SA* parent version – separation anxiety, *C-P* child version—specific phobia – environmental, *P-P* parent version—specific phobia – environmental, *SAAI* separation anxiety avoidance inventory: *C* child version, *P* parent version

These 19 papers assessed internal consistency (74%; 14/19), structural validity (63%; 12/19), convergent validity (47%; 9/19), measurement error (37%; 7/19), reliability (32%; 6/19), known-groups validity (26%; 5/19), responsiveness (16%; 3/19) and measurement invariance (5%; 1/19). No instruments assessed all COSMIN psychometric properties. No study reported on cross-cultural validity. Table 4 provides an overview of the overall level of evidence and rating of the measurement property. The following paragraphs outline the level of evidence for each of the COSMIN psychometric properties. Named measures in each section identify those with (1) sufficient ratings *and* high-quality evidence or (2) the inclusion of a study that reports on measurement development. Footnotes provide justifications for any low-quality ratings of measures.

**Table 4:**
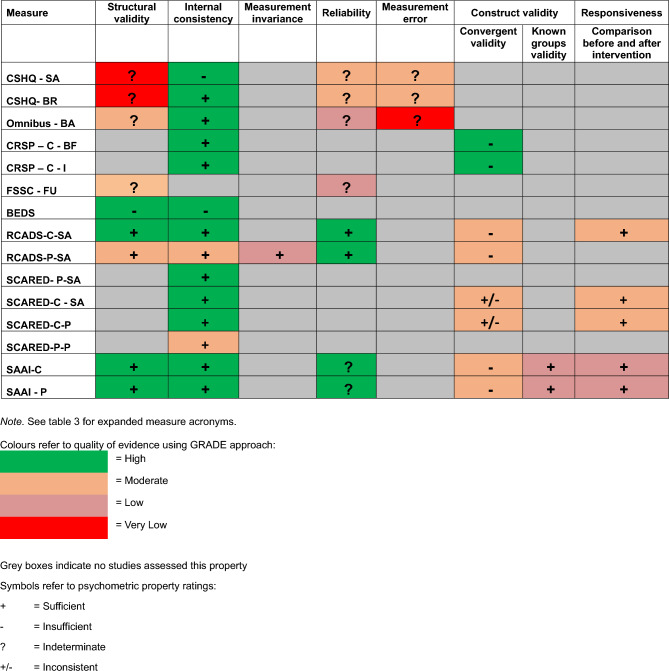
COSMIN Evaluation of Measures Psychometric Properties and Evidence Quality

### Measurement Development and Content Validity

There was only one study that reported on measurement development (Omnibus; Biggs et al., 2012). The process for the development of the Omnibus was described briefly, entailing the modification of the Children’s Sleep Habits Questionnaire (CSHQ) (Owens et al., 2000a, 2000b), Sleep Disturbance Scale for Children (SDSC) (Bruni et al., 1996) and additional items drawn from the clinical expertise of the authors were incorporated. The quality of the Patient-Reported Outcome Measure (PROM) design study of the Omnibus was of doubtful quality. The authors described a pilot study briefly, but insufficient data were reported and therefore, the COSMIN checklist could not be applied. There was no concept elicitation or content validity study conducted for any measure.

### Reliability

#### Internal Consistency

The majority of measures (i.e. subscales of interest) (93%; 14/15) had an assessment of internal consistency (except for FSSC). Of those that assessed internal consistency, more than half of the measures (57%; 8/14) indicated a sufficient rating and high-quality evidence (CRSP-C-BF, CRSP-C-IS, CSHQ-BR, Omnibus, RCADS-C-SA, SAAI-C, SAAI-P, SCARED-C-P, SCARED-C-SA, and SCARED-P-SA; see Table 3 for expanded acronyms and list of studies). Two measures (14%; 2/14) had high-quality evidence for an insufficient rating (i.e. Cronbach’s alpha < 0.70).

#### Reliability (Test–retest)

Fifty-three per cent of measures (8/15) had evidence for reliability (test–retest). The majority of measures (75%; 6/8) indicated an indeterminate rating for reliability, except for two measures (25%; 2/8) which had a sufficient rating and of high-quality evidence (RCADS-P-SA and RCADS-C-SA). The primary reason for the indeterminate ratings was the lack of reporting of intraclass correlation coefficient (ICC) or weighted Kappa, as required by the COSMIN criteria.

### Measurement Error

Three measures (20%; 3/15) reported on measurement error, all of which had indeterminate ratings. An indeterminate rating was assigned as the minimal important change (MIC) was not defined.

### Validity

#### *Construct Validity*: *Convergent*

Over half of measures (53%; 8/15) had studies reporting on convergent validity. Most ratings (75%; 6/8) were insufficient, supported by high- or moderate-quality studies. Two measures (25%; 2/8) were inconsistent and of moderate quality. A measure received an inconsistent rating when multiple studies indicated both sufficient and insufficient evaluations.

#### *Construct Validity*:

### Known Groups

Only two measures (13%; 2/15) reported on known-groups validity. Data reported indicated a sufficient rating, although evidence was of low quality.^1^

#### Responsiveness (Hypothesis Testing Before or After Intervention)

Thirty-three per cent of measures (5/15) reported on responsiveness and all measures were rated as sufficient. Three measures (60%; 3/5) were of moderate-quality evidence and two measures (40%; 2/5) were of low quality.^2^

#### Structural Validity

Structural validity was assessed by 60% of measures (9/15). There were mixed ratings across measures, with four (44%; 4/9) indeterminate ratings, four (44%; 4/9) sufficient ratings and one (11%; 1/9) insufficient rating. Only four measures (33%; 3/9) were rated as sufficient and had high-quality evidence (RCADS-P-SA, RCADS-C-SA, SAAI-C and SAAI-P). Although the majority conducted a factor analysis as per COSMIN guidelines, there were incomplete data for fit indices, leading to indeterminate ratings for forty-four per cent (4/9) of measures.

#### Measurement Invariance

Only one study (7%; 1/15) examined measurement invariance (RCADS-P-SA). This measure received a sufficient rating, although of low-quality evidence.^3^

## Stage 3: Recommendations

Table 5 provides recommendations on suitable measures for future use. No measures are recommended as most suitable as no studies demonstrated sufficient content validity. Eleven measures met criteria for potential suitability but require further validation. Four measures did not meet criteria for recommendation.

**Table 5:** Recommendations for Measures Assessing Sleep-related fears

Category	Description of category criteria	Measures
A: Most suitable	Measures with high-quality evidence for sufficient content validity (any level) and at least low-quality evidence for sufficient internal consistency	None
B: Promising but requires further validation studies	Measures that have potential but require further validation studies	• Omnibus-BA • FSSC-FU • SCARED-P-SA • SCARED-C-SA • SCARED-C-P • SCARED-P-P • SAAI-C • SAAI-P • RCADS-C-SA • RCADS-P-SA
C: Not recommended	Measures that should not be recommended (i.e. measures with high-quality evidence for an insufficient psychometric property)	• CSHQ-SA • CRSP-C-BF • CRSP-C-I • BEDS

See Table 3 for expanded measure acronyms

## Discussion

This systematic review summarised and critically evaluated the psychometric properties and quality of parent- and child-report measures assessing sleep-related fears in children, using the COSMIN checklist. Valid, reliable and responsive tools are essential for assessing early markers that may contribute to anxiety and sleep disorders. Overall, the data on the psychometric properties of these measures were mixed and incomplete, with much of the evidence ranging from low to moderate quality. There were no measures that met criteria to recommend as most suitable, highlighting the critical need for better measurement tools for sleep-related fears in children.

The first stage of this review identified the breadth of parent- and child-report measures that include an assessment of sleep-related fears in children from 7 to 12 years old. Of the 810 papers retrieved, we identified 66 papers assessing the measurement properties of 43 measures. Despite the high prevalence and impact of sleep-related fears in children, only a small portion of the total papers retrieved included measures that had any assessment of sleep-related fears. Furthermore, among the measures that did assess sleep-related fears, the evaluations were brief, with an average of two relevant items per measure. Given the heterogeneous nature of the content of these fears, a small number of items is unlikely to capture the full scope. Moreover, there were no measures with the main aim of assessing sleep-related fears.

The second stage of this review evaluated the psychometric properties and methodological quality of studies for measures that included a more comprehensive assessment of sleep-related fears (defined as 3 or more items) using the COSMIN checklist. There were 19 studies evaluating 15 parent- and child-report measures. Data were incomplete and missing, with no measures having complete data for all psychometric properties. The most commonly reported psychometric property in the COSMIN review was internal consistency (74%). The least reported psychometric properties were cross-cultural validity (0%), content validity (0%), measurement invariance (5%), and responsiveness (16%).

For reliability, most studies reported only internal consistency as an indicator. The majority of the studies (57%) reporting internal consistency had a sufficient rating and high-quality evidence. Other components of reliability, including inter- or intra-rater correlations and measurement error, were not as frequently reported and had mostly indeterminate ratings. These indeterminate ratings indicate that whilst measurements were conducted, gold standard metrics were not employed or reported, thereby impeding a full assessment of the measure’s reliability. The only measure that had an adequate and full assessment of reliability (both sufficient rating and high or moderate quality) including measurements of internal consistency, test–retest and inter-rater correlations was RCADS-SA-C and RCADS-SA-P (Ebesutani et al., 2009, 2015; Kosters et al., 2015).

Validity was most commonly evaluated through structural validity (63%) and convergent validity (47%). Most studies were allocated an indeterminate rating for structural validity. The evidence for convergent validity was insufficient or inconsistent, as results did not meet the threshold for correlations set out in the hypotheses. There was only one study that partially assessed content validity evaluated through a PROM development paper (Omnibus; Biggs et al., 2012). Other aspects important to content validity were not explored, such as relevance, comprehensiveness and comprehensibility. Content validity, described as “the degree to which the content of an instrument is an adequate reflection of the construct to be measured”, is noted as the most critical measurement property by COSMIN (Mokkink et al., 2010, p. 743). A measure with inadequate content validity can affect all other measurement properties and lead to inappropriate conclusions (Terwee et al., 2018). Irrelevant or missing concepts, indicating poor content validity, can compromise structural validity, reliability, and interpretability. Although the studies in this paper primarily focussed on reliability, a high Cronbach’s alpha or adequate test–retest reliability does not guarantee that the intended construct is fully or accurately measured. It was surprising that there was no comprehensive exploration of content validity by any study, both regarding the full-scale measure and subscales of interest for this paper.

Other psychometric properties that were not or rarely explored in papers include measurement error, responsiveness, measurement invariance and cross-cultural validity. Assessing measurement error can help better understand where the sources of error. Lower measurement error increases confidence in scores and reduces the number of participants required to detect intervention effects (Devine, 2003). Responsiveness refers to the detection of the actual change, without over- or under-estimating (Mokkink et al., 2021). When measures are used to assess longitudinal change, it is essential that they can detect clinically important changes, as determined by their responsiveness (Guyatt et al., 1989). Measurement invariance is important for ensuring that comparisons, e.g. across groups, time points or conditions, are meaningful (Chan, 2011). Similarly, cross-cultural validity is important to ensure that a measure developed in one culture is valid in another (Sperber, 2004). Poor measurement invariance and cross-cultural validity can lead to erroneous research conclusions. This review included papers with measures translated into English but excluded those that were not in English. As a result, studies focussing on cross-cultural validity may have been overlooked, highlighting an area for future reviews to explore. Nevertheless, further research on a more complete range of psychometric properties is crucial in aiding measurement selection and improving assessment of sleep-related problems.

In the third stage, the COSMIN results were reviewed against established criteria to formulate recommendations for measures for sleep-related fears in children. There were no measures that met criteria as ‘most suitable’ (i.e. measures with high-quality evidence for sufficient content validity (any level) and at least low-quality evidence for sufficient internal consistency). Nearly half of the measures showed potential but require further validation (FSSC-FU, Omnibus-BA, RCADS-C-SA, RCADS-P-SA, SAAI-C, SAAI-P, SCARED-C-SA, SCARED-C-P, SCARED-P-P and SCARED-P-SA). There were several instruments that are not recommended for use (BEDS, CSHQ-SA, CRSP-C-BF and CRSP-C-I) as there were insufficient properties with high-quality evidence.

## Strengths and Limitations

This systematic review has several strengths. It is the first to investigate the breadth and psychometric properties of sleep-related fears in children, contributing key insights to the field and expanding the existing body of knowledge in anxiety and sleep in children. The review uses rigorous approaches through its use of COSMIN methodology in reviewing psychometric properties and its broad scope in its systematic search terms, ensuring comprehensive coverage of the literature.

This study had several limitations. Whilst this systematic review adhered to the rigorous COSMIN methodology, it uses the “worst score counts” approach, which disregards higher-quality scores on other items and could be considered highly stringent. Although this approach does not account for the nuances, the authors aptly note that poor methodological aspects of a study cannot be compensated by good aspects (Terwee et al., 2018). As such, the review team opted to follow the rigorous COSMIN guidelines, despite this limitation.

Another possible limitation is that only one reviewer evaluated studies using the COSMIN methodology, which may have introduced potential biases or errors. To mitigate this, the reviewer conducted extensive piloting to increase familiarity with the COSMIN and discussed and resolved any uncertainties with the senior author throughout. Additionally, the reviewer and the broader team have a strong and highly aligned expertise in this area, which the review would have benefited from.

This systematic review aimed to focus on the use of these measures in children aged 7–12 years old. Studies were included if the mean age was between 7 and 12 years. However, it is possible that some studies excluded based on the mean age still included samples of children aged 7–12 years. Defining specific age ranges as opposed to means may have expanded coverage of relevant literature.

## Future Research

This review highlighted a scarcity of psychometrically sound and high-quality assessment tools for sleep-related fears in children. Future research would benefit from the development of rigorous and comprehensive measures with the main focus on the assessment of sleep-related fears. In addition, there were no studies that reported on all aspects of content validity. It is key for authors to develop measures using best-practice methodology and to report on the measure development process and content validity. Given the limited research on sleep-related fears in children (Lewis et al., 2021), qualitative studies may help in increasing our understanding of these heterogeneous fears and inform measure development. In addition, future research would benefit from examining non-English language sleep-related fear measures and papers.

Future psychometric studies would benefit from referring to COSMIN for guidance on study design and reporting. More studies evaluating multiple psychometric properties would significantly advance the field and provide more robust and comprehensive data for evaluation. Exploring diverse assessment modalities, including interviews and objective tools such as actigraphy and wearables, could enhance scalable and precise measurement and treatment of sleep-related concerns across the lifespan (Aji et al., 2022; Inhulsen et al., 2022).

## Conclusion

This three-stage systematic review has revealed a small proportion of existing measures assess sleep-related fears in children aged 7–12 years old. As a result, no measure can be recommended as suitable for sleep-related fears in children based on psychometric properties and study quality using the COSMIN methodology. The majority of measures require further validation studies. It is important for future research to develop more comprehensive and rigorous measures that adequately and reliably capture these heterogeneous fears, thereby improving the identification and treatment of sleep-related fears in children.

## Supplementary Information

Below is the link to the electronic supplementary material.

Supplementary file1 (DOCX 21 KB)

## Data Availability

No datasets were generated or analysed during the current study.

## Footnotes

1 This study received a low-quality rating for known-groups validity as an adequate description of characteristics and differences between the subgroups was not provided (Box 9, standard 5) and statistical methods were not optimal (only p-values and Cohen’s d were reported; Box 9, standard 6).

2 The study received a low-quality rating for responsiveness, because there was no a priori hypothesis regarding the magnitude of the effect size (Box 10, standard 12). As noted in COSMIN, a large effect size is not necessarily indicative of responsiveness.

3 The low rating for the paper was given due to (1) a lack of reporting of differences between groups (COSMIN Box 5, standard 1) and (2) unequal groups (*n* = 100 vs. *n* = 195) and a modest sample size in the context of a complex 6-factor model (COSMIN Box 5, standard 4) (Harrington, 2008; Kline, 2008).
