# Supplementary material for: Measures of Sleep-Related Fears in Children: A Systematic Review of Psychometric Properties Using COSMIN
Source: Clin Child Fam Psychol Rev. 2025 May 21;28(2):439–57. doi: 10.1007/s10567-025-00526-6 (PMC12162706; doi:10.1007/s10567-025-00526-6)
Supplement: Supplementary file 1 — Supplementary file1 (DOCX 21 KB) [file 10567_2025_526_MOESM1_ESM.docx]

**SUPPLEMENTARY INFORMATION**

**Appendix 1. Example Search Strategy (Pubmed)**

(sleep[MeSH]) OR (sleep[Title/Abstract]) OR (insomnia[Title/Abstract]) OR (insomnia[MeSH:noexp]) OR ("anxi*"[Title/Abstract] OR "anxiety"[MeSH Terms:noexp] OR "fear*"[Title/Abstract] OR "fear"[MeSH Terms:noexp] OR "phobi*"[Title/Abstract] OR "phobic disorders"[MeSH Terms:noexp])) AND

questionnaire*[Title/Abstract] OR instrument[Title/Abstract] OR scale[Title/Abstract] OR measur*[Title/Abstract] OR index[Title/Abstract] OR test*[Title/Abstract] OR Checklist[Title/Abstract] OR tool[Title/Abstract] OR inventor*[Title/Abstract] AND

(psychometrics[MeSH]) OR ("Reproducibility of Results"[MeSH]) OR ("Validation Studies as Topic"[MeSH]) OR ("Validation Study" [Publication Type]) OR (Bias[MeSH:exp]) OR ("Predictive Value of Tests"[Mesh]) OR ("Discriminant Analysis"[Mesh]) OR (Psychometric*[Title/Abstract]) OR (reliability[Title/Abstract]) OR (validity[Title/Abstract]) OR (validation[Title/Abstract]) OR (reproducibility[Title/Abstract]) OR (bias[Title/Abstract]) AND

 infant*[Title/Abstract] OR child*[Title/Abstract] OR pediatric*[Title/Abstract] OR youth[Title/Abstract] OR school*[Title/Abstract] OR preschool[Title/Abstract] OR pre-school[Title/Abstract] OR (Child[Mesh])
